# Particle System Based Adaptive Sampling on Spherical Parameter Space to Improve the MDL Method for Construction of Statistical Shape Models

**DOI:** 10.1155/2013/196259

**Published:** 2013-06-05

**Authors:** Rui Xu, Xiangrong Zhou, Yasushi Hirano, Rie Tachibana, Takeshi Hara, Shoji Kido, Hiroshi Fujita

**Affiliations:** ^1^Graduate School of Medicine, Yamaguchi University, Tokiwadai 2-16-1, Ube, Yamaguchi 755-8611, Japan; ^2^Graduate School of Medicine, Gifu University, Yanagito 1-1, Gifu 501-1194, Japan; ^3^Information Science and Technology Department, Oshima National College of Maritime Technology, Komatsu 1091-1, Oshimagun Suooshimacho, Yamaguchi 742-2193, Japan

## Abstract

Minimum description length (MDL) based group-wise registration was a state-of-the-art method to determine the corresponding points of 3D shapes for the construction of statistical shape models (SSMs). However, it suffered from the problem that determined corresponding points did not uniformly spread on original shapes, since corresponding points were obtained by uniformly sampling the aligned shape on the parameterized space of unit sphere. We proposed a particle-system based method to obtain adaptive sampling positions on the unit sphere to resolve this problem. Here, a set of particles was placed on the unit sphere to construct a particle system whose energy was related to the distortions of parameterized meshes. By minimizing this energy, each particle was moved on the unit sphere. When the system became steady, particles were treated as vertices to build a spherical mesh, which was then relaxed to slightly adjust vertices to obtain optimal sampling-positions. We used 47 cases of (left and right) lungs and 50 cases of livers, (left and right) kidneys, and spleens for evaluations. Experiments showed that the proposed method was able to resolve the problem of the original MDL method, and the proposed method performed better in the generalization and specificity tests.

## 1. Introduction

Statistical shape models (SSMs) are an efficient method which considers statistical information of a set of training shapes to improve the robustness of medical image processing algorithms. It has been widely used in the task of segmentation and achieved good results [1]. It is reported that the best three ranked segmentation algorithms on livers are based on SSMs in the contest held in 2007 [2]. According to our knowledge, several SSMs have been proposed, which are point distribution models (PDMs) [3], m-rep [4], SPHARMs [5, 6], and so on. We focus on PDMs in this paper since it is the most widely used SSMs.

The main problem of the construction of SSMs is how to determine corresponding points on each shape. For simple 2D shapes, that is, faces, corresponding points can be determined manually by placing a set of landmarks on shapes; however obvious disadvantages are both large manual labors and the inevitably subjects' bias. Especially for complex 3D shapes of clinical data, manual determination of hundreds or thousands of landmarks is not practical. Therefore, lots of researches have focused on automatic ways to find corresponding points [1]. The determination of shapes' correspondence can be generalized as a shape registration problem. A group-wise based method is proposed to align a group of shapes according to an information-based theory, called minimal description length (MDL) [7], in order to get the simplest description of constructed SSMs [8]. It is reported that the MDL based method is the state-of-the-art method according to exhaustive evaluations [9]. At first, the MDL method can only find the corresponding points of 2D shapes [8, 10, 11]. Recently, it is generalized to deal with the problems of 3D shapes [12–14]. We follow the pilot study [12], since the open source codes helped us to understand its essence [15]. In the following context, we call the pilot study [12] the original MDL method. 

The original MDL method can be divided into three steps. First, each original surface mesh of genus-0 topology is initially mapped onto a parameter space of a unit sphere by a conformal mapping [16]. Each vertex of an original surface mesh is transformed onto the unit sphere, and a new spherical mesh built by transformed vertices according to their original linked relationships is called a parameterized mesh in this paper. Second, these parameterized meshes are aligned by manipulating their vertices simultaneously on the unit sphere in order to minimize the MDL measure. Finally, each of the aligned parameterized meshes is sampled by a set of uniformly distributed points on the unit sphere, and the sampled points are mapped back by the corresponding inverse conformal mapping onto each original shape surface to obtain corresponding points. Mapping surfaces onto a unit sphere makes shapes' registration become easier, since the freedoms of translation and scale can be eliminated and only the freedoms of rotation and subjects' differences are left. However, the manipulation of parameterized meshes makes them to be distorted during MDL registration. Some triangles of parameterized meshes are shrunk while others are expanded. This makes the predetermined conformal mapping become poor. If uniformly distributed positions are sampled on the unit sphere for each parameterized mesh, less sampling points are obtained in the shrunk regions and more sampling points are obtained in expanded regions. Therefore, this method has suffered from the problem that determined corresponding points are usually densely located in some parts and coarsely in other parts on original surfaces.

This problem may be resolved by either redetermining a new conformal mapping for each shape to replace the poor one or finding adaptive sampling positions on the unit sphere instead of the uniformly distributed sampling method. In this paper, we use the latter method to resolve the problem of the original MDL method. Here, we propose a method to obtain adaptive sampling positions on the unit sphere by considering distortions of parameterized meshes. The proposed method is based on a particle system which is originally adopted for modeling isosurfaces of shapes in [17]. 

## 2. Materials and Methods

**Figure 1:**
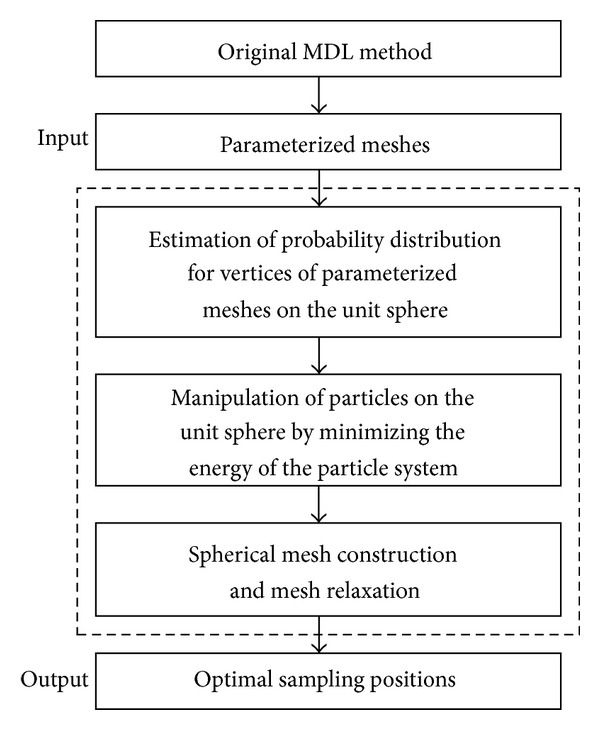
The flowchart of the particle system based adaptive sampling on spherical parameter space to improve the MDL method. The main steps of the proposed method are surrounded by the dash rectangle.

The proposed particle-system based method is operated when the original MDL registration is finished. Therefore, the proposed method can be seen as a postprocessing of the pilot study [12]. The flowchart of the proposed method is given by Figure 1. The input is a set of parameterized meshes aligned by the original MDL method [12] on the unit sphere. Vertices of these meshes are used to generate a probability distribution to show the frequency of vertices existing at a position on the unit sphere. This probability distribution can reflect how parameterized meshes are distorted in the MDL registration. Next, a set of particles are uniformly placed on the unit sphere. A particle can be seen as a dot or a sampling position. Each particle gives repulsive forces to push its surrounding particles away from itself. The value of the force for a pair of particles is related to both of their distance and the probability of vertices at which the particles are located. An energy function can be calculated by adding the forces of all particles. By minimizing this energy, particles are manipulated on the unit sphere until the energy becomes steady. The final particles are treated as vertices to build a spherical mesh. Finally, this mesh is relaxed to slightly adjust vertices' positions on the spherical surface, and the optimal sampling points are the vertices of the relaxed mesh. When the proposed method is finished, each parameterized mesh is sampled at these sampling points on the unit sphere, and then the sampled points are transformed back to each surface of organs by its corresponding inverse conformal mapping to get corresponding points of each shape. The details are described in the following subsections.

### 2.1. Probability Distribution for Vertices of Parameterized Meshes

We adopt the probability distribution for vertices of parameterized meshes located on the unit sphere to describe how these parameterized meshes are distorted in the original MDL registration. If triangles of parameterized meshes are shrunk in a region, there will be more vertices in that part. Therefore, vertices have a high probability to exist there. Conversely, the probability is low in regions where triangles are expanded. We adopt a parzen window [18] based method to estimate this probability distribution. Given **v**_*ij*_ denoted by the *j*th vertex of the *i*th parameterized mesh, the probability of vertices existing in a location **x** on the unit sphere *p*(**x**) can be calculated by

(1) $$\begin{array}{c} p\left(x\right)=\alpha \sum_{i}\sum_{j}\varphi \left(||x-v_{ij}||\right), \end{array}$$

where *α* is a coefficient to ensure ∫*p*(**x**)*d* **x** = 1 and ||**x** − **v**_*ij*_|| is the Euclidian distance of the two points **x** and **v**_*ij*_. *φ*(*d*) is a trunked Gaussian kernel whose definition is given by

(2) $$\begin{array}{c} \varphi \left(d\right)=\frac{1}{\sqrt{2\pi }\sigma }\exp\left(-\frac{d^{2}}{2\sigma^{2}}\right)\;d\leq 3\sigma ,\\ \varphi \left(d\right)=0\;d>3\sigma , \end{array}$$

where *σ* is set to be 0.033 in this paper.

**Figure 2:**
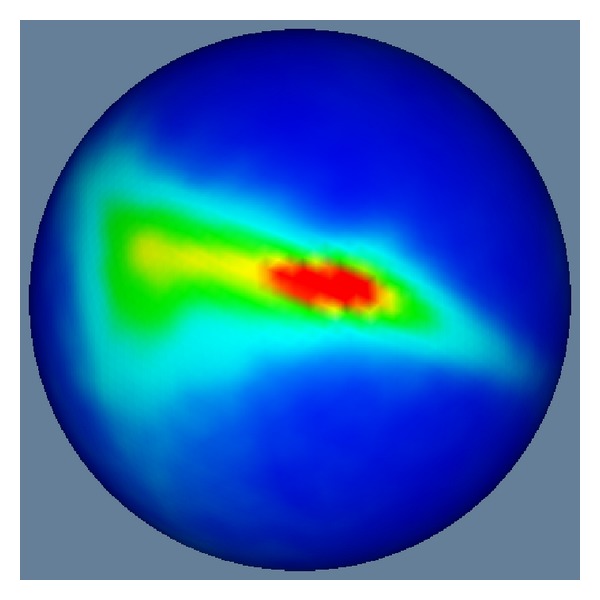
An example of the estimated probability distribution for vertices of parameterized meshes on the unit sphere. The red regions show where probabilities of vertices existing in those regions are high, and the blue regions show where the probabilities of vertices existing in those regions are low. The estimated probability distribution is able to reflect how triangles of parameterized meshes are distorted. For example, high probability (red color) means that triangles in those regions are shrunk, and low probability (blue color) means that triangles in those regions are expanded.

In implementation, we discretize the unit sphere by a 5-time recursively-refined icosahedron mesh which contains 10242 vertices uniformly distributed on the unit sphere. An example of the estimated probability distribution is shown by Figure 2. 

### 2.2. Energy of the Particle-System

The energy of the particle-system is defined by

(3) $$\begin{array}{c} E\left(x_{1},\ldots ,x_{N}\right)=\sum_{i=1}^{N}\sum_{i\neq j}\omega \left(x_{i},x_{j}\right)\cdot F\left(x_{i},x_{j}\right), \end{array}$$

where **x**_1_,…, **x**_*N*_ are all *N* particles and their energy is denoted as *E*(**x**_1_,…, **x**_*N*_). *F*(**x**_*i*_, **x**_*j*_) is the force term of *i*th and *j*th particles and *ω*(**x**_*i*_, **x**_*j*_) is the weight associated for them. We adopt the force term suggested in [17]. Its definition is given by

(4) $$\begin{array}{c} F\left(x_{i},x_{j}\right)=\{\begin{array}{cc} \text{cot}\left(\frac{||r_{ij}||}{\sigma }\cdot \frac{\pi }{2}\right)+\frac{||r_{ij}||}{\sigma }\cdot \frac{\pi }{2}-\frac{\pi }{2} & ||r_{ij}||\leq \sigma ,\\ 0 & ||r_{ij}||>\sigma , \end{array} \end{array}$$

where ||**r**_*ij*_|| is the Euclidean distance of the pair of particles which are **x**_*i*_ and **x**_*j*_. *σ* is a parameter. Larger value of *σ* means that a particle has taken forces from more surrounding particles in a larger region, so more computation costs are required. But the value should be large enough to make particles move. In our system, we choose it to be 0.25 for trade-offs between performances and computation costs.

It is important to design suitable weight terms *ω*(**x**_*i*_, **x**_*j*_) to make the particle system work properly. If all weight terms are set to be equal, particles finally spread uniformly on the unit sphere when the energy is minimized. Here, we make use of the estimated probability distribution of vertices to design suitable weight terms in order to obtain the optimal sampling positions which are adaptive to distortions of parameterized meshes. The definition of the weight terms *ω*(**x**_*i*_, **x**_*j*_) is given by

(5) $$\begin{array}{c} \omega \left(x_{i},x_{j}\right)=\frac{D\left(x_{i}\right)+D\left(x_{j}\right)}{2},\\ D\left(x_{i}\right)=\{\begin{array}{cc} \frac{a}{{\left(p(x_{i})\right)}^{\gamma }}, & p\left(x_{i}\right)>b,\\ \frac{a}{b}, & p\left(x_{i}\right)\leq b, \end{array} \end{array}$$

where **x**_*i*_ is the position on which the *i*th particle is located. *p*(**x**_*i*_) is the estimated probability of vertices at the location of **x**_*i*_. *a*, *b*, *γ* are parameters whose values are set to be 1*e* − 5, 1*e* − 5 and 2.2, respectively in experiments.

### 2.3. Minimization of the Energy of the Particle-System

The optimal positions of particles are obtained by minimizing the energy defined in (3). This can be generalized as an optimization problem. Considering the number of particles is large, we adopt an iterative gradient descent based method to make it converge faster to a local minimum. Here, we only update one particle's position along the opposite gradient direction at one time, and this procedure is operated for each particle in turns. One iteration is called when the iterative updating for all particles is finished. The pseudocode is given by Algorithm 1. Its initialization requires a set of uniformly-distributed particles on the unit sphere (**x**_*i*_^0^ (*i* = 1,2,…, *N*)), the same maximal step length associated for each particle for gradient descent (*λ*_*i*_ = *λ*_*Max*⁡_ (*i* = 1,2,…, *N*)), the minimal step length (*λ*_*Min*⁡_),  and the total iteration times (*T*). When we update the *i*th particle's position in the *t*th iteration, we firstly calculate a position **y**′ using the current step length *λ*_*i*_ for the *i*th particle along the opposite gradient direction. By using the condition of ||**y**′|| = 1, we ensure that the new position **y** is on the surface of unit sphere. Then, we calculate the new energy *E*_new_, by replacing **x**_*i*_^*t*^ with the new position **y**. If *E*_new_ is smaller than the former energy *E*_old_, we accept **y** as the new position for the *i*th particle in the *t*th iteration, update *E*_old_ by *E*_new_ and end the loop. If not, we halve the step length of the *i*th particle and try a new position to see whether it can minimize the energy. However, the step length should not be smaller than *λ*_*Min*⁡_. In such a case, we give up updating the position of the *i*th particle in the *t*-iteration and only let the step length of the *i*th particle to be equal to *λ*_*Min*⁡_.

In out experiments, we set the iteration times *T* to be 15. *λ*_*Max*⁡_ and *λ*_*Min*⁡_ are determined as follows. Before Algorithm 1 begins, we randomly choose 10 percent of particles and calculate the mean value of the norms (or lengths) of their gradients. This mean value is denoted by ${\bar{g}}_{\mathrm{norm}}$. *λ*_*Max*⁡_ is set to be $10.0/{\bar{g}}_{\mathrm{norm}}$, and *λ*_*Min*⁡_ is set to be $1e-4/{\bar{g}}_{\mathrm{norm}}$.

### 2.4. Construction of Sampling Mesh and Relaxation

When the Algorithm 1 is finished, the obtained particles' positions are treated as vertices to be linked by Delaunay triangles to build a spherical mesh. This mesh gives how sampling points (particles) are linked with each other, and this linked information is required in SSMs. Additionally, we require this information to slightly adjust particles on the spherical surface to obtain optimal sampling positions. According to [19], Delaunay triangles of a surface can be obtained by constructing convex hulls for a set of 3D points. Constructing triangles directly by this way lead to a locally ununiform mesh. This can be improved by the technique of mesh relaxation, which slightly adjusts vertices' positions according to their linked information. Here, we adopt the method suggested by [20] to optimize the constructed mesh. Finally, the positions for the vertices of the relaxed mesh are the optimal sampling positions on the unit sphere.

## 3. Results and Discussion

### 3.1. Data

We evaluate the proposed method by 6 kinds of human organs, which are livers, left and right lungs, left and right kidneys, and spleens. We collect 47 cases of lungs and 50 cases of other organs from different subjects by a practical clinical protocol using high-resolution CT (HRCT) with the slice thickness of 1 mm and in-plane resolution around 0.6 mm. The organs' regions are manually labeled on original scans and then resampled to be saved as binary masks with the resolution of  1 mm  ×  1 mm  ×  1 mm. Finally, marching cubes followed by mesh decimation using the visualization toolkit (VTK) [21] is adopted to get a triangles' mesh for each organ. The number of vertices for each mesh is related to organ's sizes. There are about 3000–5000 vertices for livers and lungs and 1000–2000 vertices for kidneys and spleens. These meshes are used as inputs for evaluations.

### 3.2. Optimal Sampling Positions Obtained by the Proposed Method

**Figure 3:**
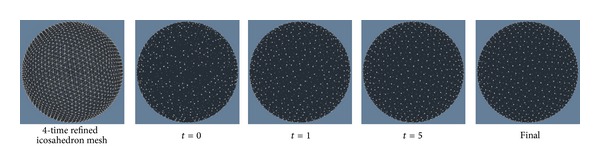
Example to obtain 1000 uniformly distributed points on the unit sphere by the iterative procedure of Algorithm 1. Here, we set the term *ω*(**x**_*i*_, **x**_*j*_) = 1 in (3). We randomly choose 1000 vertices of a 4-time iteratively refined icosahedron mesh as initial points (*t* = 0). Particles' positions are given after the 1st (*t* = 1) and 5th (*t* = 5) iterations are finished. The final result is shown at last.

Algorithm 1 requires a given number of uniformly distributed points (particles) on the unit sphere for its initialization. It looks like a strict and difficult condition; however, we can obtain these points with any given number if we make a little change on Algorithm 1 by setting the terms of *ω*(**x**_*i*_, **x**_*j*_) in (3) to be an equal value (i.e., *ω*(**x**_*i*_, **x**_*j*_) = 1). For example, we can get 1000 points uniformly distributed on the unit sphere as follows. Here, we make use of a 4-time iteratively refined icosahedron mesh which contains 2562 uniformly distributed vertices on the unit sphere. First, we break the mesh and randomly select 1000 vertices as initialized positions for particles. Then we set the terms *ω*(**x**_*i*_, **x**_*j*_) to be 1 and run Algorithm 1. When the iterative procedure is finished, we can obtain 1000 points uniformly distributed on the unit sphere. This procedure is illustrated by Figure 3. Actually other methods could also be used for the initialization, such as random selection 1000 points on the unit sphere. 

**Figure 4:**
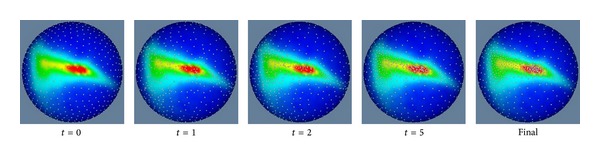
Iterative procedure to manipulate 1000 particles on the unit sphere according to Algorithm 1, where the weight terms of *ω*(**x**_*i*_, **x**_*j*_) in (3) are set to be (5). The probability distribution for vertices of parameterized meshes is shown with colors on the unit sphere. Red parts show where the probability is high, and blue parts show where the probability is low. The figure (*t* = 0) shows initialized particles which are uniformly distributed on the unit sphere. Particles' positions are shown after the 1st (*t* = 1), 2nd (*t* = 2), and 5th (*t* = 5) iterations are finished, respectively. Final positions of particles are shown by the most right figure from which it can be seen that more particles are located in red regions and less particles are located in blue regions.

The uniformly distributed points are treated as initial positions for the particle system to get optimal sampling positions. By setting the weight terms *ω*(**x**_*i*_, **x**_*j*_) in (3) to be (5), Algorithm 1 is able to make the final particles' locations to be adaptive to the probability of vertices of parameterized meshes.  Figure 4 gives examples to show the iterative procedure to manipulate 1000 particles on the unit sphere according to Algorithm 1. 

**Figure 5:**
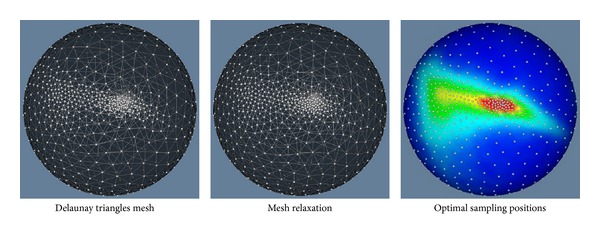
Results of constructed Delaunay triangles mesh, mesh relaxation, and the obtained 1000 optimal sampling positions on the unit sphere.

When the iterative procedure is finished, the obtained particles are treated as vertices to build a spherical mesh by Delaunay triangles. The left figure in Figure 5 gives the constructed Delaunay triangles mesh on the unit sphere. It can be seen that triangles in local regions are not uniform. The mesh is improved by mesh relaxation, shown in the middle figure in Figure 5. The final optimal sampling positions overlaid on the probability distribution of vertices are shown in the right figure of Figure 5.

### 3.3. Corresponding Points and Evaluations on SSMs

**Figure 6:**
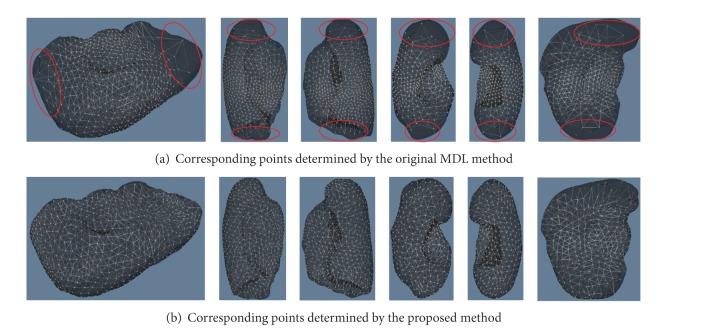
Corresponding points determined by the original MDL method [12] and the proposed method. The organs from left to right are livers, right lungs, left lungs, right kidneys, left kidneys, and spleens. Red circles illustrate the regions where there are fewer corresponding points.

We evaluate the performances of the proposed method and the original MDL method by using six kinds of human organs. Examples of corresponding points determined by the two methods are given by Figure 6. It can be seen that corresponding points are gathered in partial regions on the surfaces of organs for the results of the original MDL method. Especially, there are nearly no corresponding points in the regions indicated by red circles. This problem is able to be corrected by the proposed method. It can be seen that corresponding points are almost uniformly located on the surfaces of organs.

We build two SSMs based on the corresponding points determined by the two methods and compare their performances by the generalization and specificity tests, which are the most widely used method to evaluate SSMs suggested by [22]. Generalization tests are based on leave-one-out experiments, where *N* − 1 shapes are used for training SSMs and the remaining one is used to test whether it can be represented by the trained SSMs. The distance between the untrained shape and the reconstructed shape by SSMs is calculated to show how much difference exists. Low values indicate that the constructed SSMs have good generalization performances on untrained shapes. The measure of generalization is defined by

(6) $$\begin{array}{c} G\left(M\right)=\frac{1}{N}\sum_{i=1}^{N}\text{Dis}\left(X_{i},X_{i}^{\prime }\right), \end{array}$$

where *N* is the total number of shapes, **X**_*i*_ is the *i*th untrained shape, **X**_*i*_′ is the corresponding reconstructed shape by SSMs using the largest *M* eigen-vectors, and Dis(**X**_*i*_, **X**_*i*_′) is the distance of the two shapes, **X**_*i*_ and **X**_*i*_′.

In specificity tests, the constructed SSMs are used to synthesize a large number of shapes. Each synthesized shape is compared with training shapes and finds the closest one. The mean value of distances between synthesized shapes and their corresponding closest training shapes is used as the measure of specificity tests. Low values indicate that the trained SSMs have good specificity on training shapes. The measure of specificity is defined by

(7) $$\begin{array}{c} S\left(M\right)=\frac{1}{L}\sum_{i=1}^{L}\underset{X_{j}\in \Omega_{X}}{\min}\text{Dis}\left(Y_{i},X_{j}\right), \end{array}$$

where *L* is the number of synthesize shapes by SSMs using *M* largest eigen vectors, **Y**_*i*_ is the *i*th synthesize shape, *Ω*_**X**_ is the set of training shapes to build SSMs, and Dis(**Y**_*i*_, **X**_*j*_) is the distance of the two shapes, **Y**_*i*_ and **X**_*j*_. In experiments, we synthesize 10000 shapes for the specificity test.

Shape distances in generalization and specificity tests are calculated by (8), in order to make the comparison of two shapes not to be concentrated on corresponding points [23].

(8) $$\begin{array}{c} \text{Dis}\left(X,Y\right)=\frac{1}{2}\left[\frac{1}{U}\sum_{x\in X}\underset{y\in Y}{\min}\;d\left(x,y\right)+\frac{1}{V}\sum_{y\in Y}\underset{x\in X}{\min}\;d\left(x,y\right)\right], \end{array}$$

where **x** and **y** are vertices of the two shape **X** and **Y**, respectively, *d*(**x**, **y**) is the Euclidian distance of two vertices, and *U* and *V* are the number of vertices for the two shapes.

Since livers and lungs are larger than kidneys and spleens, we use 1000 corresponding points to build SSMs of livers and lungs and 500 corresponding points to build SSMs of kidneys and spleens. Additionally, we adopt general-purpose computing on graphics processing units (GPGPU) to deal with the large computation costs on (8).

**Figure 7:**
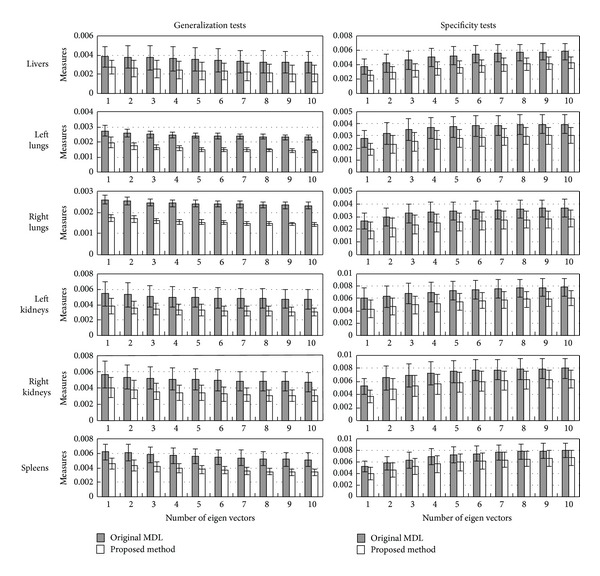
Two SSMs constructed by both the original MDL method [12] and the proposed method are compared in the generalization and specificity tests on the human organs of livers, left and right lungs, left and right kidneys, and spleens. We compare the two SSMs when the largest *M* (1~10) eigen vectors are used. The bars give the mean values, and the positive and negative steps give the standard deviations.

Results on generalization and specification tests are given in Figure 7. We calculate the mean values and the standard deviations for generalization and specificity measures to compare the two methods. It can be seen that the proposed method gives smaller measures of generalization and specificity for all the six kinds of organs, which means that the proposed method performs better than the original MDL method.

### 3.4. Discussion

Although it is a good idea to conformally transform each shape onto a unit sphere to eliminate variations on translations and scales before the MDL registration is performed [12], the conformal mappings become poor because manipulation of parameterized meshes makes them to be distorted in shape alignment. Therefore, a uniform distributed sampling on the parameterized meshes after the MDL registration causes the determined corresponding points to be densely located in some parts and coarsely in other parts. An example of uniform sampling adopted in the original MDL method is shown by the most left figure in Figure 4 (with subtitle “*t* = 0”). It can be seen that there are a few sampling positions located in regions where triangles of parameterized meshes are shrunk (red regions) and relatively more sampling positions in regions where triangles are expanded (blue regions). This causes the problem that determined corresponding points are not uniformly distributed on the original surfaces of organs.

In the proposed method, the obtained optimal sampling positions are adaptive to the distortions of parameterized meshes. The most right figure (with the subtitle “Final”) in Figure 4 gives such an example. It can be seen that there are a lot of sampling positions in the red regions where triangles are shrunk and relatively few positions in the blue regions where triangles are expanded. Therefore, determined corresponding points can spread uniformly on the original surfaces of organs even though conformal mappings become poor after MDL registration.

**Figure 8:**
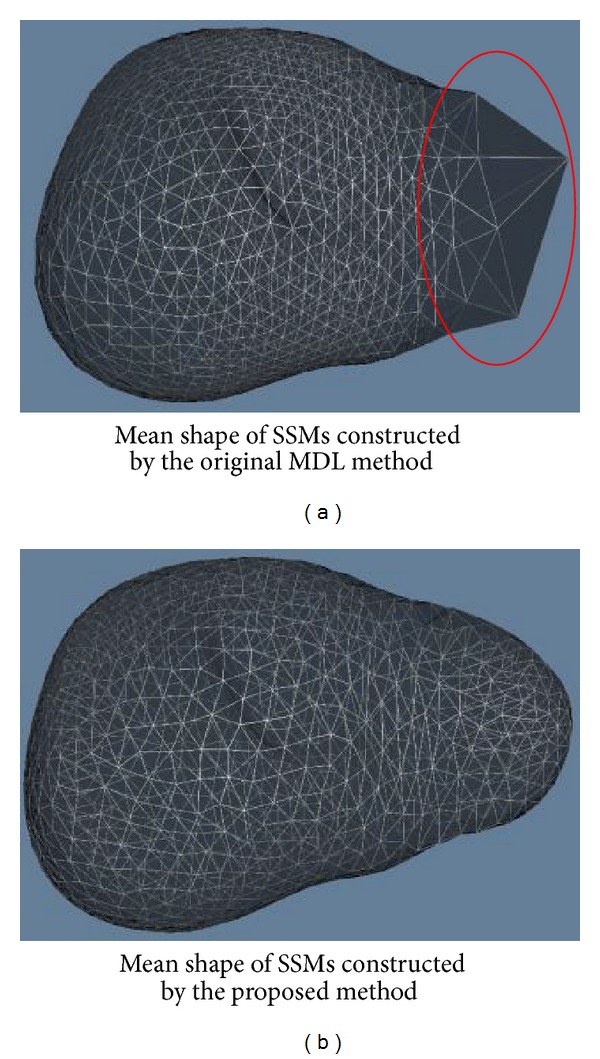
Mean shapes of SSMs constructed by the corresponding points determined by the original MDL method [12] and the proposed method for livers. It can be seen that the part indicated by the red circle cannot represent detailed shapes of livers.

In the generalization and specificity tests, it can be seen that SSMs built by corresponding points determined by the proposed method perform better. Here, we give an example to show the reason.  Figure 8 gives the mean shapes of SSMs built by the corresponding points determined by the original MDL method and the proposed method for livers. It can be seen that the mean shape built by the original MDL method loses detailed information of livers' shapes. In particular, the mean shape decays in the part indicated by the red circle. However, the mean shape contains more details for the livers' shapes by using the proposed method. Therefore, SSMs built by the proposed method performs better than SSMs built by the original MDL method.

**Figure 9:**
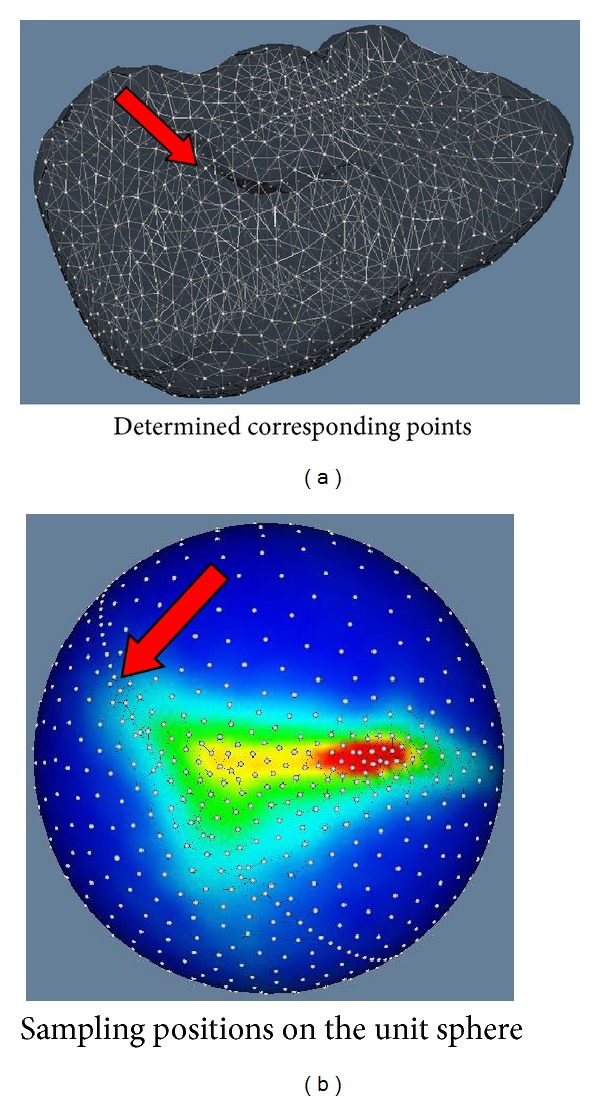
An example to show the dense-line artifact we encountered when we reimplemented the work [24]. (a) The determined corresponding points for a case of livers. The dense line artifact is indicated by the red arrow. (b) The adaptive sampling positions on the unit sphere. The line artifact exists in the reconnected part (indicated by the red arrow) through which two half-spherical sampling meshes are united.

It seems that there is another work to resolve the same problem of original MDL in [24], where a 2D-dithering based remeshing method was adopted [20, 25]. We reimplement it; however we encounter a dense-line problem. An example of this problem is shown by Figure 9. In methodology, the method [24] has to firstly get two adaptive half-spherical meshes separately and then unit them to construct a whole spherical mesh whose vertices are the optimal sampling positions. If the densities of vertices on the two meshes' margins (through which the two meshes are connected) are different, this artifact problem will happen. We refer to all the related literature [20, 23, 24], and we find no descriptions of how to keep the two densities equal while ensuring the required condition of the MDL method that the number of sampling positions should be unchanged. Although it seems that there is no such a line artifact problem in [24], we do not know how to avoid this problem. In the proposed method, since we manipulate a set of particles to find optimal sampling positions directly on the unit sphere, the connection of two meshes is not required. Therefore, the proposed method is able to avoid the line artifact problem in methodology. Additionally, we feel that it could not be fair to compare the performances of the proposed method and the reimplemented version with dense-line artifacts. Therefore, we only state the fact that we encounter in the reimplementation of [24] and do not give the compared results.

Additionally, we note that there is a particle and entropy based method for SSMs [26]. The difference is that we apply the particle-based method as the postprocessing step of MDL method in order to obtain optimal sampling positions on the unit sphere.

## 4. Conclusion

MDL based shape registration method proposed in [12] was a state-of-the-art method to determine the corresponding points on surfaces of 3D organs. Since uniformly distributed points on the unit sphere were used to sample the shapes registered in the parameter space, the obtained corresponding points were not uniformly distributed on the original surfaces of organs. In this paper, we proposed a particle system based method to find the optimal sampling positions on the unit sphere to resolve this problem. In our method, a set of particles was manipulated on the unit sphere to find the optimal sampling positions by minimizing a carefully designed energy function. We evaluated the proposed method on six kinds of human abdominal and chest organs, which were livers, left and right lungs, left and right kidneys, and spleens. We collected 47 cases of lungs and 50 cases of other organs from different subjects to evaluate the proposed method. Experiments showed that the proposed method was able to find optimal sampling positions on the unit sphere and resolve the problem of the original MDL method. Additionally, we compared the proposed method with the original MDL method in the generalization and specificity tests. Experimental results showed that SSMs built by the proposed method performed better than SSMs built by the original MDL method. In future, we will apply the built SSMs in some segmentation tasks of human organs.

## Figures and Tables

**Algorithm 1:**
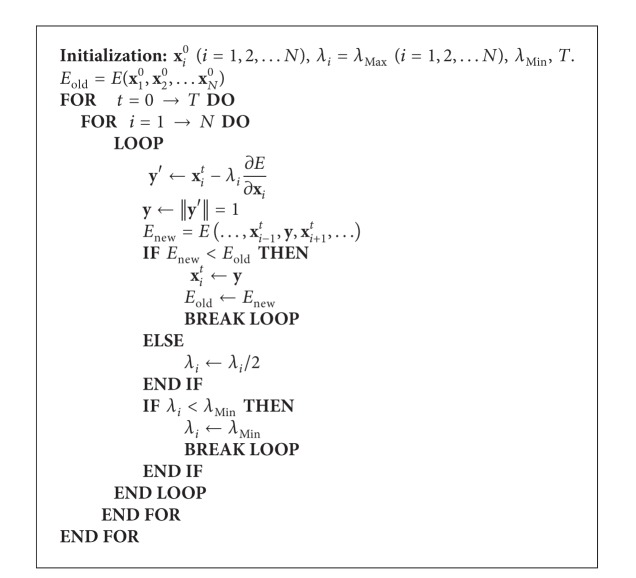
An iterative gradient descent based method to minimize the energy of the particle system defined in (3).
